# Analysis of Face Milling of Hard Steel 55NiCrMoV7 by Studying Rough and Semi-Finished Machining and the Influence of Cutting Parameters on Macroscopic Chip Dimensions

**DOI:** 10.3390/ma17143434

**Published:** 2024-07-11

**Authors:** Claudiu Ionuţ Malea, Eduard Laurenţiu Niţu, Daniela Monica Iordache, Ştefan Lucian Tabacu, Aurelian Denis Negrea, Claudiu Bădulescu

**Affiliations:** 1 Regional Research-Development Center for Innovative Materials, Processes, and Products for the Automobile Industry (CRC&D-Auto), National University of Science and Technology POLITEHNICA Bucharest, 060042 Bucharest, Romania; claudiu_ionut.malea@upb.ro (C.I.M.); aurelian.negrea@upb.ro (A.D.N.); 2 Faculty of Mechanics and Technology, National University of Science and Technology POLITEHNICA Bucharest, 060042 Bucharest, Romania; daniela.c.iordache@upb.ro (D.M.I.); stefan.tabacu@upb.ro (Ş.L.T.); 3 Dupuy de Lôme Research Institute (IRDL)—UMR CNRS 6027, ENSTA Bretagne, F-29200 Brest, France; claudiu.badulescu@ensta-bretagne.fr

**Keywords:** hard milling, chip temperature, cutting force, chip morphology, response surface methodology, ANOVA

## Abstract

Hard milling is being increasingly used as an alternative to EDM due to its high productivity. The present paper presents the results of theoretical-experimental research on the face milling of hard steel 55NiCrMoV7. A comprehensive analysis of cutting temperatures and forces during single-tooth milling and a morphological examination of the resulting chips are conducted for roughing and semi-finishing operations. The temperature is analyzed in the chip formation area, and the detached chips and the cutting force are analyzed through their tangential, radial, and penetration components, depending on the contact angle of the cutter tooth with the workpiece. The analysis of chip morphology is carried out based on the dimensional and angular parameters of chip segmentation and their degree of segmentation. Based on the central composite design and the response surface method, it is shown that it is possible to mathematically model the dependence of the macroscopic dimensions of the detached chips on the cutting parameters. The determined process functions, the maximum chip curling diameter, and the maximum chip height allow for establishing the influence of the cutting parameters’ values on the chips’ macroscopic dimensions and, thus, guiding the cutting process in the desired direction.

## 1. Introduction

Hard machining is widely used in many manufacturing industries, and the dies and molds industry is the main application area. Hardened metals, tool steels, nickel-based alloys, chromium-based alloys, titanium-based alloys, etc., are considered hard metals [1,2]. Hard steels are considered to have a hardness above 45HRC, and extra-hard steels are those with a hardness above 55HRC [1,3].

The hard machining of metals can be carried out by different processes, such as turning [4,5,6], milling [7], or drilling [8]. Classic technologies for processing parts with complex surfaces made of hard metals use electro-erosion processes, followed by grinding processes. The current trend of modern processing technologies with high productivity is to use hard cutting processes in machining centers, especially milling centers, followed by grinding processes with rotary tools applied to the same technological processing systems [2].

The milling of hard metals is used to obtain complex surfaces [9,10], profiled surfaces [11,12,13], or flat surfaces [7,14]. Face milling is widely used in processing dies and molds, especially at the level of contact surfaces, where very narrow flatness conditions of the order of micrometers are usually necessary.

The strategies for removing the allowance material in the machining of hard and extra-hard metals have in mind finishing, semi-finishing, or roughing processes; this is determined from the perspective of the rigidity of the machine tools or the processing conditions prescribed for the parts to be made. For this reason, process forces are one of the most important parameters in the application and research of hard metal cutting processes. They can be measured in the measuring system using stationary dynamometers [15,16,17,18] or in the machining system using rotary measuring devices [9,19,20,21]. In the study of milling processes, force analysis can be performed globally, on the entire cutting process [22,23], locally, for a complete rotation of the tool [24], or on the formation of a single chip [25,26]. Günay et al. [27] studied the influence of the parameters of the cutting regime on the resulting cutting force when milling hard steel in dry environments and concluded that the feed per tooth has the biggest influence on it. Cui et al. [28] analyzed the steel milling process with a hardness of 47HRC and found that minimum cutting forces are obtained at a cutting speed of 200 m/min, and they increase substantially at speeds above 1400 m/min.

Due to the high intensity of the plastic deformation processes in the shearing area of the processed material, as well as the tribological ones that take place at the semi-finished product-tool-chip interfaces, the amount of heat released in the process of cutting hard metals is very high [13,29]. This heat is distributed between the part, the tool, and the chip, with most of the heat being absorbed by the chips. The temperature at the tool-chip interface is high and negatively influences tool wear, being the main process parameter affecting its cutting edges [13].

Temperature measurement in milling processes is frequently performed using thermographic techniques or thermocouples. The thermographic technique allows the determination of the temperature at the cutting tool level [30,31,32] and the measurement of the maximum temperatures of the released chips [22,33]. Thermocouples are used to measure the temperatures in the contact areas between the tool and the part [24,34]. The variation in the values of the cutting regime parameters has an important influence on the process temperatures. Liu et al. [35] showed that in hard metal milling processes, the process temperatures vary with the variation in the cutting width and the tooth feed, even if the other parameters are kept constant. Cheng et al. [36] measured temperatures in the milling process of a low-machinability material with thermocouples and concluded that the cutting speed has the most significant influence on the process temperature.

The chip formation process comprises a phase of plastic deformation, followed by a chip initiation and breakage phase, as described in various scientific papers [12,37,38,39]. Niu et al. [37] presented the general theories of chip formation, highlighting the fact that the model of shear planes (adiabatic shear theory) is suitable for processing materials with good plasticity. In contrast, the periodic brittle fracture theory model is suitable for processing more fragile materials. For hard machining processes, it is accepted that the chip formation model is that of the shear planes [38], and the microscopic shape of the detached chips has the appearance of some saw teeth. In the study by Zhang, S. and Guo, Y.B. [11] regarding the milling of steel with a hardness of 50HRC, it was concluded that the cutting speed and the feed per tooth are the most important parameters in the formation of chips. Their combination leads to the formation of different types of chips, from continuous chips to serrated chips. Furthermore, in connection with the milling of some hard steels, Cui, X. and Zhao, J. found that [7], for cutting speeds up to 400 m/min, the chips are formed as a series of saw teeth; however, with the increase in speed cutting that speeds up to 2000 m/min and even higher values, these chips segment and become interrupted.

Analysis of the morphology of the generated chips represents another way of investigating the mechanisms and intensity of the cutting processes because it provides measurable parameters that allow for the indirect determination of the degree of wear of the tools or the quality of the processed surfaces. In the analysis of chip morphology, a series of methods and laboratory techniques are used, which can be optical [13,40] or electronic [41,42,43]. The main elements analyzed regarding chip morphology are the radius of curling, the height of the chips formed [44], the shear angle [45], the bulge angle, the tooth pitch between shear planes, the chip peak, and the chip valley [12]. Furthermore, the color of the chips represents an evaluation indicator of the cutting process. The heat taken by the chip and the temperatures it reaches determine the different degrees of oxidation of the removed material [11], therefore, also different colors, indirectly providing information about the cutting process.

Experimental-statistical methods are frequently used to establish the dependence of some results of the hard metal milling process on the cutting parameters, such as the forces and temperatures in the process [6,46,47]. In these studies, DOE or Taguchi [4,48] are used for planning the technical experiments, which allow for a statistical analysis of the results (ANOVA) and the presentation of the mentioned dependencies in the form of response surfaces (RSM) [49,50].

From the studied scientific papers, it has emerged that there is close interdependence between process temperatures and cutting forces, which influence tool wear [26], as well as the morphology of detached chips and the roughness of the machined surface [35]. From the point of view of chip detachment, its dimensions should be as small as possible so that the cutting effort is small and the contact area with the tool tooth is small to reduce the intensity of tool wear but also to ensure an easy evacuation of the chip without safety risks. Although this aspect of the macroscopic dimensions of the detached chips is very important, in the analyzed works, no results were identified that mathematically model the dependence of the macroscopic dimensions of the chips according to the parameters of the cutting regime and, thus, allow their appropriate choice.

Considering the previously mentioned conclusions, a correlated analysis of temperatures and forces in the cutting process with chip morphology and cutting regime was deemed necessary. Thus, this study presents the results of theoretical-experimental research on the face milling process of hard steel, 55NiCrMoV7, frequently used in the construction of dies and molds. A new cutting insert is used, recommended by the manufacturer for cutting hard metals with roughing and semi-finishing regimes. The study involves (i) a detailed analysis of chip and process temperatures, cutting forces, and chip morphology for roughing and semi-finishing operations, and (ii) a determination of process functions that express the relationship between macroscopic chip dimensions and cutting regime parameters for a wide range of cutting regimes, from roughing to semi-finishing.

## 2. Experimental Procedure

### 2.1. The Workpiece Material

The study’s material is the high-alloy steel 55NiCrMoV7. Its chemical composition, determined with a Spectro Midex M (Kleve, Germany) energy-dispersive X-ray fluorescence spectrometer, is shown in Table 1.

**Figure 1:**
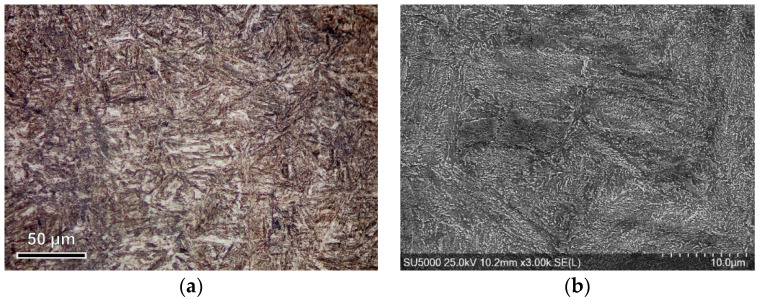
Microstructural analysis of the base material: (**a**) optical micrograph; (**b**) scanning electron microscopy (SEM).

The metallographic structure of this alloy steel, Figure 1a, is specifically created through a heat treatment process involving quenching and tempering. This process results in a fine acicular structure, which is a characteristic of the martensite created during quenching and tempering. The martensite grains are relatively uniform in size, strongly elongated, and evenly distributed without a preferential orientation, ensuring the good isotropy of the material. Additionally, very fine particles of carbides alloyed with chromium, molybdenum, and vanadium are uniformly dispersed in the martensite matrix, Figure 1b.

The initial hardness of the 55NiCrMoV7 steel was determined according to I.S. EN ISO 6507-01:2018 [51] and I.S. EN ISO 6507-02:2018 [52], based on ten measurements with a INNOVATEST FALCON 500 (Maastricht, The Netherlands) microhardness tester using a force of 1 kgf. The ultimate tensile stress and relative elongation were determined according to ISO 6892-1:2009(E) [53], based on the tensile test at ambient temperature with a strain rate of 2.22·10^−4^ s^−1^, performed on an INSTRON 5969 (High Wycombe, United Kingdom) tensile testing machine. These mechanical properties are noted in Table 2.

The complex structure, with hard phases and fine and well-controlled granulation, determines the remarkable mechanical performances of this steel: high hardness (477.4 HV) and high mechanical strength (1371.7 MPa), and the relative elongation of 7.1% suggests a good balance between hardness and ductility.

Due to its chemical composition, metallographic structure, and mechanical properties, this steel is used in applications that require high wear and shock resistance, such as molds, dies, or forging tools.

### 2.2. Work Scheme and Experimental Stand

**Figure 2:**
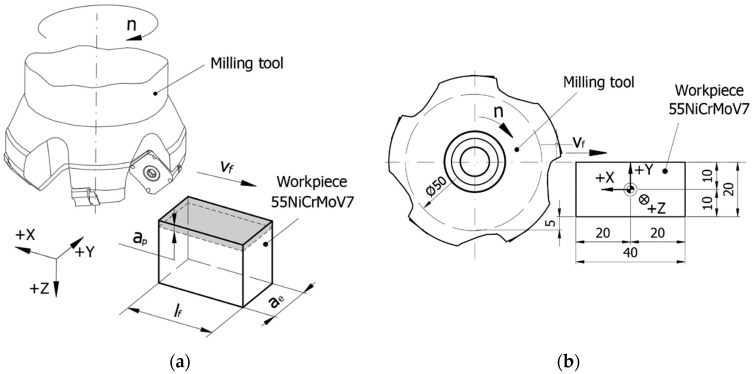
Scheme of the face milling process: (**a**) general scheme; (**b**) scheme with the positioning of the tool in relation to the semi-finished product.

The scheme of the face milling process is presented in Figure 2a. The rotation axis of the tool is positioned in the plane of penetration of the tool teeth into the semi-finished product. The dimensions of the semi-finished product, Figure 2b, were established as follows: the length of the machined surface l_f_ = 40 mm to ensure milling in the stability zone of the process, and the cutting width a_e_ = 20 mm to allow the study of the evolution of the cutting forces and temperatures during the formation of a chip, avoiding the possibility of having two tool teeth in the chip simultaneously, but also being 40% of the active diameter of the tool. These dimensions were the same for all experiments.

**Figure 3:**
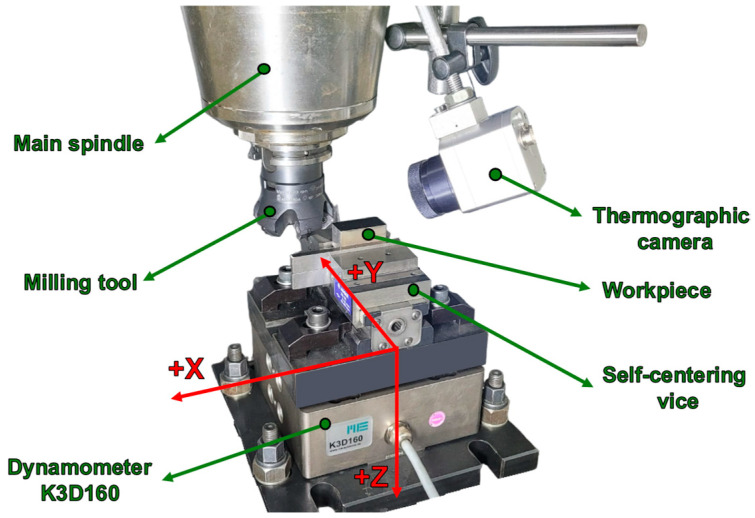
The experimental stand was used for experiments.

The machining was carried out on a numerically controlled DMG MORI ecoMill70 (Leonberg, Germany) machining center. A base plate was mounted on the machine-tool table, on which the dynamometer for measuring the cutting forces was oriented and fixed (Figure 3). The semi-finished product was oriented and fixed in a self-centering vise, mounted using an intermediate plate on the force measuring device. It was positioned so that the center coincided with the center of the dynamometer (Figure 2b).

The tool used was a Sandvik Coromant Ø50 mm 5-tooth, 45° entering angle modular milling cutter: milling head 345-050Q22-13H and indexable inserts 345R-1305M-PM 1130. The base material of the insert was a W-Co alloy, 1130 grade; the substrate was an HC carbide; and the coating was from AlTiCrN, made by physical vapor deposition (PVD). The choice was based on the favorable results regarding their use in the processing of hard steels presented in the literature [27,54,55].

### 2.3. Measuring Temperatures in the Process

The thermography technique was used to measure temperatures in the process using the Optris PI400i (Berlin, Germany) thermal imaging camera, with a measurement accuracy of ±2 °C and a thermographic image acquisition frequency of 80 Hz. Since the chip takes up most of the heat released in the cutting process, the observation area was established so that, on the one hand, the chip formation process could be analyzed and, on the other hand, it could be thermo-graphically analyzed for as many of the released chips as possible.

The thermographic camera calibration process involved the following steps:

1. A steel sample was heated to 500 °C using electric resistance.
2. During heating, the following were recorded simultaneously:
  - Thermographic images from one surface of the heated sample (40 mm × 20 mm) using an Optris PI400i thermal imaging camera.
  - Temperature values of the half surface of the sample using a calibrated K-type thermocouple, the NI9210 acquisition module, the cDAQ-9171 uni-modular platform, and Matlab R2023 software from the Analog Input Recorder.
3. The emissivity data recorded by the thermographic camera were correlated with the temperature values measured with the thermocouple.

The thermographic images were processed with Matlab R2023 software to determine the maximum temperature of the detached chips. The maximum temperature value was identified for each image, consisting of a matrix with 288 rows and 382 columns, where each coordinate was a temperature value. All the data from a perimeter of nine rows and nine columns were selected around it. Then, the average of these values was determined, representing the maximum value retained for the analyzed image. With the values thus obtained, evolution graphs of the maximum temperatures in the chips were plotted for each experiment.

### 2.4. Measuring the Cutting Force

**Figure 4:**
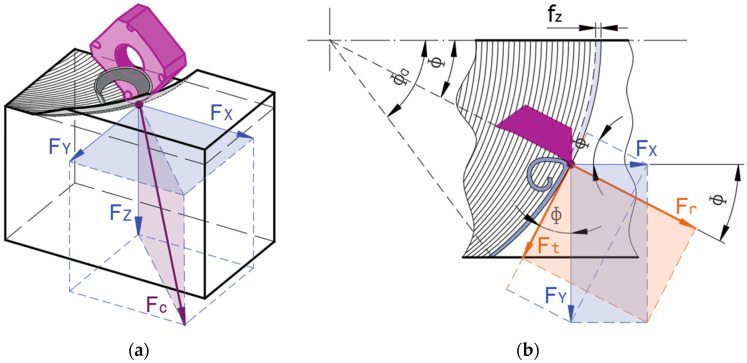
Cutting forces: (**a**) scheme of cutting forces in the measurement system; (**b**) scheme of the cutting forces in the tool machining system.

To measure the cutting force, a dynamometer with a strain gauge, Meßsysteme-model K3D160 (Hennigsdorf, Germany), with a maximum measurement limit of ±20 kN and a precision of ±0.02 kN, was used. It allowed the measurement of the components of the cutting force in the measurement system X, Y, and Z (Figure 4a), whose resultant was the cutting force Fc.

The acquisition of the force measurement data was achieved with the help of the National Instruments NI9237 acquisition module, cDAQ-9171 uni-modular platform, using Matlab R2023 software from the Analog Input Recorder module. A data acquisition frequency of 10.000 Hz was used to analyze in detail the evolution of the forces in the formation of each chip.

According to the processing scheme used, the tool progressively enters/leaves the chip; there is a zone of stability of the process in the middle area of the semi-finished product, where the insert of the tool cuts over the entire chip width. From the central stability zone of the process, the following were used: (i) the maximum values from 10 successive complete rotations of the tool (50 values = 5 teeth × 10 rotations) to determine the maximum values of the cutting force components in the measurement system; (ii) values from a complete tool rotation for detailed analysis of forces on a tool insert.

The cutting force Fc was determined as the result of the three measured component forces *F_X_*, *F_Y_*, and *F_Z_*:

(1) $$F_{c}=\sqrt{{F_{X}^{2}+F}_{Y}^{2}+F_{Z}^{2}}$$

For the analysis of the evolution of the force when cutting by a single tool tooth, the cutting force determined in the measurement system was decomposed into the specific components of the tool machining system: *F_t_* —the tangential force, in the direction of the cutting speed; *F_r_* —the radial force, in the direction of the tool radius; *F_p_* —the penetration force, in the axial direction of the tool, Figure 4b. The connection between the components of the cutting force in the tool machining system and those in the measurement system is given by the relations:

(2) $$\left\{\begin{array}{c} F_{t}\;(\Phi )=-F_{X}\;sin(\Phi )+F_{Y}\;cos(\Phi )\\ F_{r}\;(\Phi )=F_{X}\;cos(\Phi )+F_{Y}\;sin(\Phi )\\ F_{p}\;(\Phi )=F_{Z}\;(\Phi ) \end{array}\right.$$

where the angle represents the position of the tool tooth in relation to its entry into the chip.

### 2.5. Performed Experiments and Activities

The experiments were carried out in two stages, as follows:

I. The purpose of the first stage was the detailed and comparative analysis of chip and process temperatures, cutting forces, and chip morphology for roughing and semi-finishing operations. We conducted experiments using the cutting parameters recommended by the tool manufacturer. For roughing, the parameters were a_p_ = 2 mm, f_z_ = 0.2 mm/tooth, and v_c_ = 90 m/min, and for semi-finishing, the parameters were a_p_ = 1 mm, f_z_ = 0.1 mm/tooth, and v_c_ = 150 m/min.

During these experiments, the following was achieved: (1) measuring temperatures in the process and in the chips; (2) measuring cutting forces; and (3) analyzing chip morphology using optical microscopy techniques such as an OLYMPUS SZ61 (Hamburg, Germany) optical microscope and scanning electron microscopy (SEM) with a HITACHI SU5000 (Ichige, Japan) electronic microscope.

II. In the second stage of the research, we aimed to find out how the cutting parameters affected the size of the chips. We conducted experiments using a centered composite design (CCD, see Table 3). The levels of variation of the cutting parameters were a_p_ = 1/1.5/2 mm, f_z_ = 0.1/0.15/0.2 mm/tooth, and v_c_ = 90/120/150 m/min. We selected these ranges to study a wide range of cutting regimes, from roughing (experiment 2) to semi-finishing (experiment 7).

For all CCD experiments, the macroscopic dimensions of the chips were measured by optical microscopy using an OLYMPUS SZ61. The aim was to establish the relationship between the size of the chips and the cutting parameters. To achieve this, the response surface method and the ANOVA statistical analysis method were employed.

## 3. Results and Discussions

### 3.1. Analysis of the Geometric Shape of the Chips during Roughing and Semi-Finishing Milling

**Figure 5:**
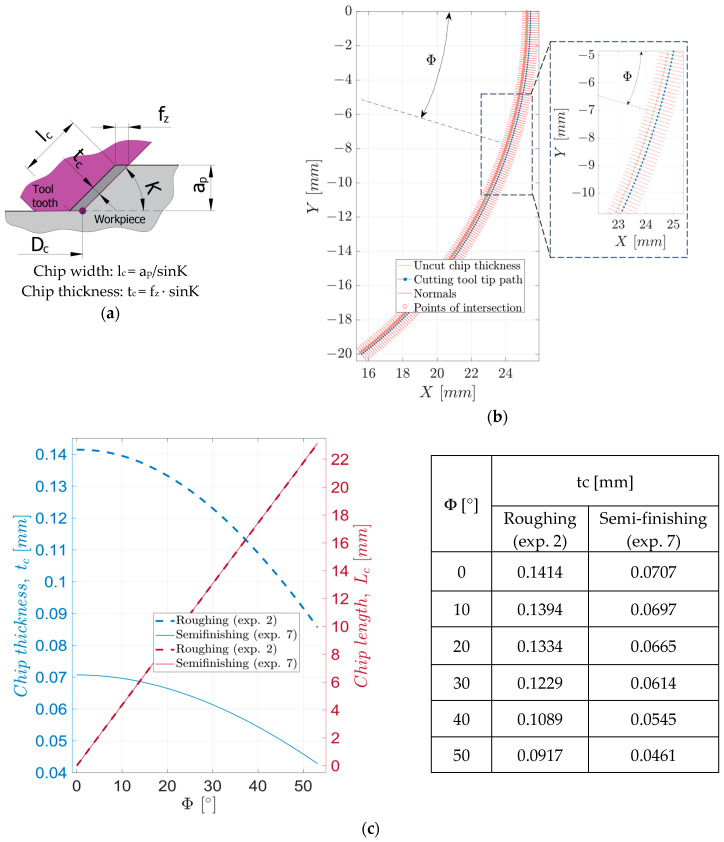
Uncut chip parameters: (**a**) section in the normal plane on the cutting speed; (**b**) numerical determination of chip thickness during roughing (exp. 2); (**c**) variation of chip thickness during roughing (exp. 2), respectively, semi-finishing (exp. 7).

When cutting the semi-finished product, the material addition is removed as chips. The detached chips’ dimensions differ from those of the uncut chips. The uncut chip, specific to the face milling process, is characterized by the following parameters, Figure 5a: chip width, l_c_, chip thickness, t_c_, and chip length, L_c_. The chip width depends on the cutting depth ap and the entering angle K, and the chip thickness depends on the feed per tooth f_z_ and the entering angle K. The length of the chip depends on the diameter of the tool, D_c_, and the cutting width, a_e_.

The thickness of the uncut chip varies depending on the position of the tool tooth in contact with the semi-finished product, a position determined by the contact angle Φ. To determine it, the trajectories traveled by the tip of two successive teeth of the tool were drawn, as shown in Figure 5b, using the relation:

(3) $$\left\{\begin{array}{c} X=\frac{D_{C}}{2}cos\Phi +f_{z}\cdot z_{c}\cdot \frac{v_{c}\cdot 1000}{\pi {\cdot D}_{c}}\\ Y=\frac{D_{C}}{2}sin\Phi \end{array}\right.\;,\;\Phi =\left(0,{sin}^{-1}\frac{a_{e}}{D_{C}/2}\right)$$

The variation of the uncut chip thickness depending on the angle Φ and the length of the chip, as shown in Figure 5c, was determined numerically as the distance between the two trajectories in the normal direction to the trajectory of the first tooth of the tool. The evolutions of the uncut chip thickness are similar for the two processes: there is a slight decrease in thickness at the beginning of chip formation (for angles Φ below 10°), followed by an increasingly pronounced decrease.

In the two analyzed experiments, for roughing and semi-finishing, the variation of the cutting depth a_p_ and that of the feed per tooth f_z_ led to an important variation of the parameters l_c_ and t_c_ of the uncut chip. At the same time, L_c_ remained constant in all of the experiments performed. Increasing the cutting depth and the feed per tooth from minimum values—for semi-finishing (a_p_ = 1 mm and f_z_ = 0.1 mm) to maximum values—for roughing (a_p_ = 2 mm and f_z_ = 0.2 mm) produces important variations of the uncut chip parameters: the chip width and thickness increase from minimum values for semi-finishing (l_c_ = 1.414 mm; t_c_ = 0.0707 mm) to maximum values for roughing (l_c_ = 2.828 mm; t_c_ = 0.1414 mm), double compared to first.

**Figure 6:**
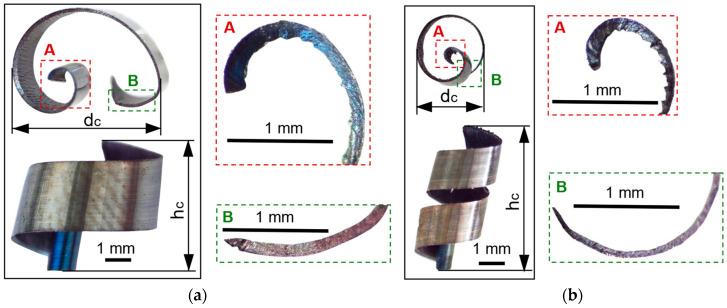
Optical micrographs of detached chips: (**a**) roughing; (**b**) semi-finished.

For the macroscopic analysis of the detached chips, their optical micrographs were made, as shown in Figure 6. The areas marked with A represent the beginning of the chip, and the areas marked with B represent the end of the chip.

The optical micrograph analysis of the detached chips reveals the following: (i) the detached chips have a spiral shape; (ii) the thicknesses of the detached chips are variable, decreasing along them from the beginning (area A) to the end (area B); (iii) the macroscopic dimensions of the chips in the two experiments are very different. It is found that the chip resulting from roughing, as shown in Figure 6a, has a greater thickness, a greater diameter, and a lower height compared to the semi-finished one, as shown in Figure 6b. Therefore, for the dimensional characterization of chips on a macroscopic scale, two-dimensional parameters of the detached chip are defined: the maximum height h_c_ and the maximum curling diameter d_c_, which depend on the intensity of the cutting parameters.

### 3.2. Process Temperature Analysis during Roughing and Semi-Finishing Milling

The chips resulting from the two processes, roughing and semi-finishing, have different colors, as can be seen in Figure 6. These colors are correlated with the temperatures during the cutting process because the temperature at the tool-chip interface is a determining factor for the degree of oxidation [11]. In both experiments, it is noted that the existence of a more intense color—dark blue—in the area at the beginning of the chip, zone A was associated with higher temperatures compared to those at the end of the chip, zone B. At the same time, the comparison of the colors of the chips from the two experiments highlights more intense, darker colors in roughing, determined by higher temperatures during this milling process, than in semi-finishing.

**Figure 7:**
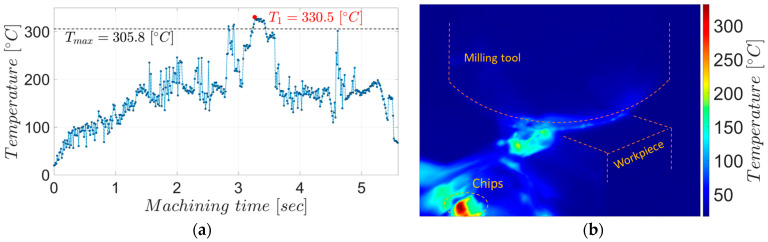
The maximum temperatures in the chips during roughing milling: (**a**) the evolution of temperatures in the chips for the entire experiment; (**b**) the thermographic image associated with point T_1_.

**Figure 8:**
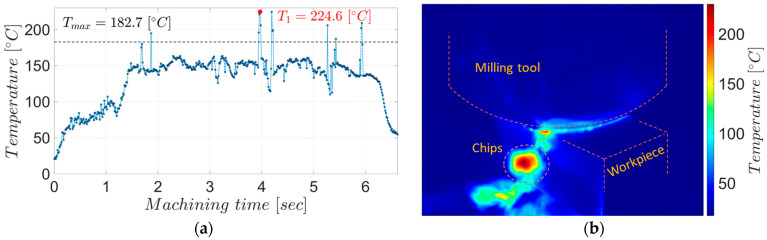
The maximum temperatures in the chips during semi-finishing milling: (**a**) the evolution of temperatures in the chips for the entire experiment; (**b**) the thermographic image associated with point T_1_.

The chips from the two experiments have different colors, which aligns with the temperature measurements showing a higher average maximum temperature during roughing, 305.8 °C, as shown in Figure 7, than during semi-finishing, at 182.7 °C, as shown in Figure 8.

As can be seen from the graphs in the two figures, the maximum temperatures measured in the detached chips gradually increase with the penetration of the tool into the material, reaching maximum values in the stability zone of the process. Since not all chips detached during the milling process could be observed (due to the low acquisition frequency of the thermal imaging camera compared to the tool rotation frequency), the maximum values within an experiment were averaged in the areas where the chips could be observed.

**Figure 9:**
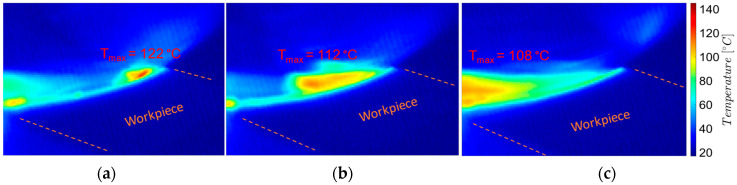
Thermographic images from the milling process near the tool-semi-finished contact area, roughing: (**a**) the beginning of the process—chip formation; (**b**) middle of the process; (**c**) the end of the process—chip detachment.

**Figure 10:**
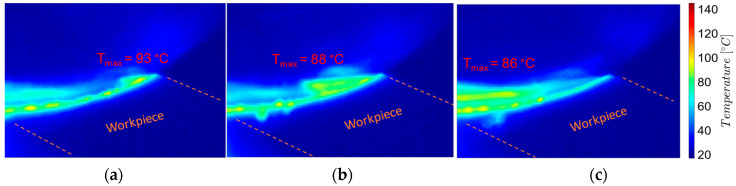
Thermographic images from the milling process near the tool-semi-finished contact area, semi-finishing: (**a**) the beginning of the process—chip formation; (**b**) middle of the process; (**c**) the end of the process—chip detachment.

To analyze the temperatures during the milling process, images were taken and processed near the chip formation zone: the beginning of the chip formation process (the entry of the tool tooth into the chip), the middle zone of the uncut chip, and the end of the chip detachment process (exit of the tool tooth from the chip), as shown in Figure 9 and Figure 10.

From the analysis of the images in Figure 9 and Figure 10, it follows that, on the one hand, there is a decreasing evolution of the temperatures in the chip formation zone, from the beginning of the chip formation to its detachment, and, on the other hand, the temperature values during the roughing are higher (by 20–30 °C) than those from semi-finishing. These temperature variations are correlated with the intensity of the mechanical deformation processes in the shear zone of the processed material and the amount of heat produced by them.

The variation of the uncut chip thickness, with the decrease in thickness from the beginning of the formation towards the chip detachment, makes the process of mechanical deformation in the shear zone of the processed material more intense at the beginning of the formation of a chip, producing a greater amount of heat than at the end. The highest temperatures at the beginning of the chip formation are correlated with the very small variations of the uncut chip thickness: up to 2 μm in the case of roughing—which corresponds to a maximum uncut chip length of 2 mm and an angle Φ of 10°, respectively, up to 1 μm in the case of semi-finishing, which corresponds to a maximum uncut chip length of 1 mm and an angle Φ of up to 10°.

Similarly, increasing the uncut chip width lc—as in the case of roughing (when ap = 2 mm and lc = 2828 mm) compared to semi-finishing (when ap = 1 mm and lc = 1414 mm)—intensifies the mechanical deformation in the area of shearing of the processed material, producing a greater amount of heat.

### 3.3. Analysis of Cutting Force during Roughing and Semi-Finishing Milling

**Figure 11:**
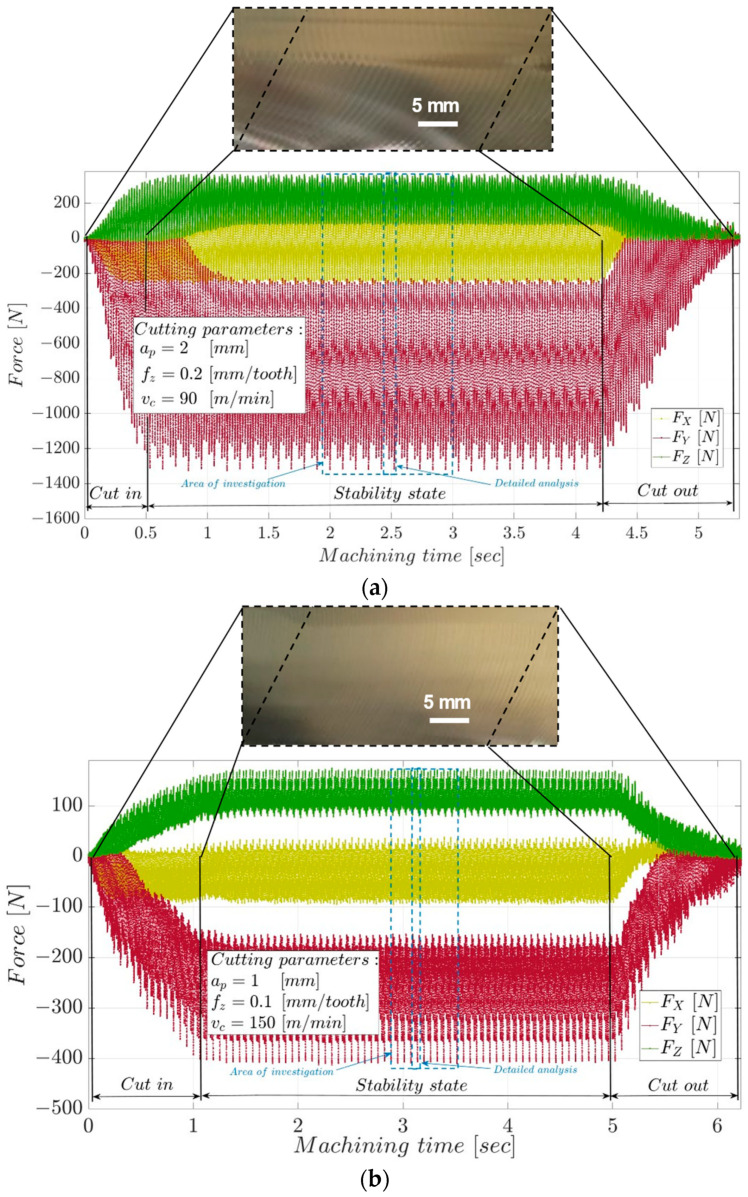
The evolution of cutting forces in the measurement system: (**a**) roughing; (**b**) semi-finished.

The evolution of the cutting forces in the measurement system during the milling process is shown in Figure 11. The three areas specific to the face milling process are highlighted: the chip entry zone, where there is a rapid increase in the components of the cutting forces as the cutting width a_e_ increases from zero to the maximum value of 20 mm; the stability zone of the process, in which the tool cuts successively with the five teeth on a maximum cutting width, and the components of the cutting force have minor variations; the chip exit zone, where the chip width gradually decreases from the maximum value to zero, and the components of the cutting force decrease. The absolute maximum values of the cutting force F_c_ are 1293.5 N in the case of roughing and 390.13 N in the case of semi-finishing.

The variation of the uncut chip section and the temperature during chip formation influence the evolution and size of the cutting forces. The double values of the dimensions of the uncut chip section during roughing compared to those during semi-finishing determine a 4-fold increase in the detached chip section, leading to a sharp increase in the mechanical and thermal processes that take place during material removal. If the intensification of mechanical processes leads to an increase in cutting forces, the increase in temperature facilitates the cutting process by decreasing the material’s resistance to deformation. Thus, it is found that the ratio between the absolute maximum value of the cutting force F_c_ during roughing and that during semi-finishing is 3.3.

**Figure 12:**
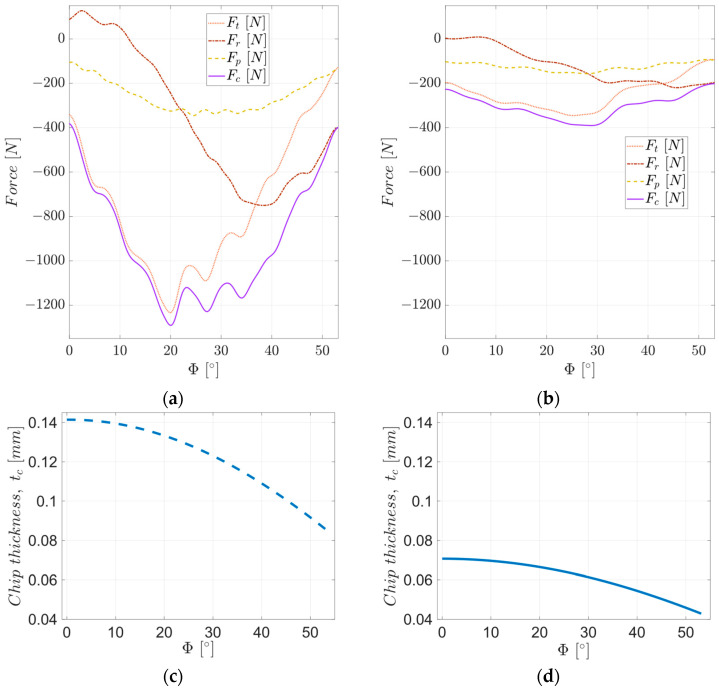
The evolution of the cutting force components in the tool machining system: (**a**) roughing; (**b**) semi-finishing; and chip thickness evolution for (**c**) roughing and (**d**) semi-finishing.

To analyze the evolution of the cutting force during cutting by a single tooth of the tool, the components of the cutting force F_t_, F_r_, and F_p_ were analyzed in the tool machining system. Figure 12 shows the evolution of the average values of the components of the cutting force for a complete rotation of the tool (average values on the five teeth of the tool).

These evolutions of the components of the cutting force are correlated with the intensities of the mechanical deformation processes in the shear zone of the machining material and of the tribological ones that take place at the tool-chip and tool-part interfaces. The working scheme used, in which the chip formation starts from the maximum to the minimum thickness (see Figure 12c,d) and the very small variation of the uncut chip thickness at the beginning of the cutting, have the effect of carrying out the process with maximum intensity cutting in its first part.

The tangential cutting force F_t_ is mainly determined by the effort required to deform the machining allowance and produce its separation around the tool edge. As it follows from Figure 12, the values of this force are high from the beginning of the chip formation and increase up to a maximum value, corresponding to the contact angles of the tool tooth, Φ, 20° for roughing, respectively, 25° for semi-finishing. Up to these angles, the thickness of the uncut chip decreases very little (up to 0.008 mm for both processes—see Figure 12c,d), the mechanical deformation process in the shear zone of the chip is very intense, causing an increase in the deformation energy of the material, and the heat released is important. With the increase in the contact angle of the tool tooth above the value associated with the maximum tangential force, the amount of heat released in the semi-finished product-tool-chip contact area becomes important. The uncut chip’s thickness decreases dramatically, leading to a decrease in the intensity of the mechanical deformation process in the shear zone of the chip and, thus, a decrease in the tangential cutting force.

The radial force F_r_ is determined by the friction between the tool and the blank (at the beginning of the process), by the friction between the material removed from the blank (the chip being formed) and the tool (its rake face), and by the plastic deformation process through which the chip is formed (around the rake face of the tool). The work scheme determines its slightly positive values at the beginning of the process when it acts perpendicular to the advance direction and by tool-workpiece friction. These values decrease as the size of the tool tooth contact angle increases, becoming negative, and the absolute value increases to a maximum value lower than that of the maximum tangential force. The contact angle of the tool tooth at which the radial force is maximum is greater than the one at which the tangential force is maximum, in the case of both experiments: 38° for roughing and 45° for semi-finishing. This aspect is determined by the fact that the effort required to form the chip (the plastic deformation of the chip) is long, and its decrease occurs towards the end of the cutting, with the increase in the temperature of the formed chip.

The penetration force F_p_ is determined by the friction between the tool and the machined surface (the resulting part) and has a relatively uniform evolution. The value from which this force starts is the same for the two experimental cases studied. However, during roughing, there is a much more pronounced curvature than during semi-finishing. This curvature is determined by the elastic deformation of the experimental system and its dynamic behavior, which is also visible in the evolution of the other two cutting force components.

From the previous analysis, it follows that the tangential cutting force, F_t_, has a major role in the formation and removal of the chip and is the main component of the cutting force, F_c_, essentially determining its evolution. The radial cutting force, F_t_, acts simultaneously in the feed direction and the direction perpendicular to the feed direction, depending on the contact angle of the cutter tooth, tending to push the tool sideways. It has an important contribution to the cutting force F_c_ and the stability of the milling process. The penetration force, F_p_, is oriented along the tool axis, pushing the tool into the workpiece, and can affect tool wear on the clearance face. The use of very different values for the parameters of the cutting regime, associated with the roughing and semi-finishing processes, respectively, leads to significantly different values of the maximum values of the cutting forces.

### 3.4. Analysis of Chip Morphology during Roughing and Semi-Finishing Milling

Chip morphology refers to the shape and size of the chips, along with the structural characteristics of the material in the chip. This provides important information regarding the material separation mechanism and the integrity of the machined surface, being a means of assessing the performance of the milling process [13,56].

**Figure 13:**
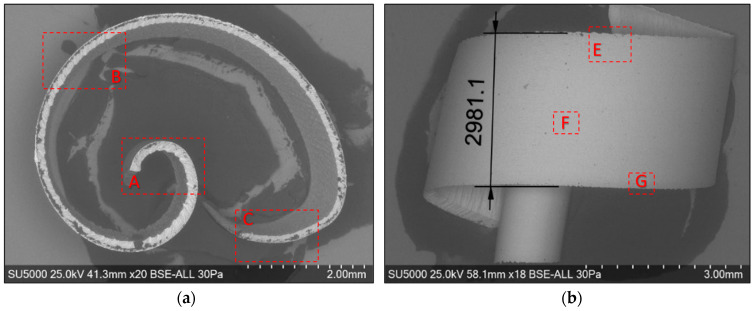
Overall SEM micrographs of the chip detached during roughing (exp. 2), highlighting the detailed areas: (**a**) front view; (**b**) side view.

**Figure 14:**
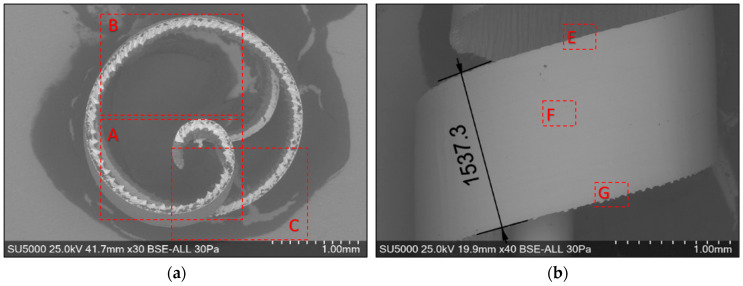
Overall SEM micrographs of the detached chip at semi-finishing, highlighting the detailed areas: (**a**) frontal view; (**b**) side view.

**Figure 15:**
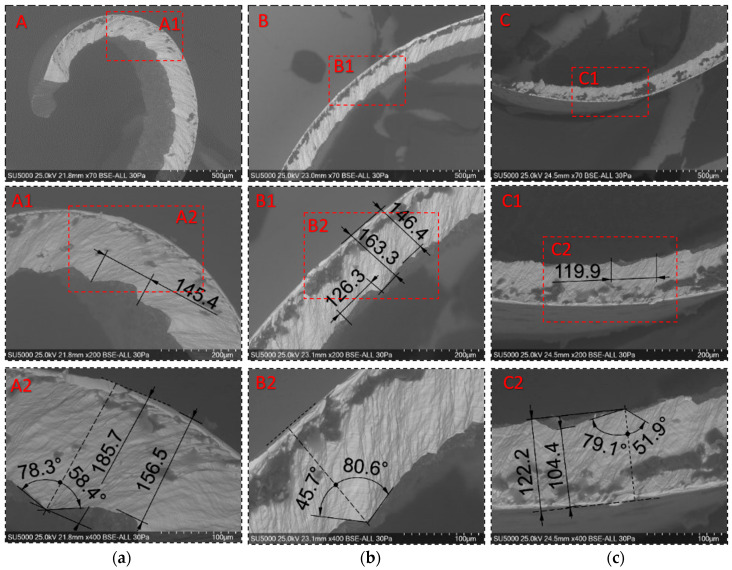
Detailed SEM micrographs of the chip detached during roughing (exp. 2) with different degrees of magnification of the areas specific to the front part of the chip: (**a**) the starting area of the chip (A,A1,A2); (**b**) the middle area of the chip (B,B1,B2); (**c**) the chip end zone (C,C1,C2).

**Figure 16:**
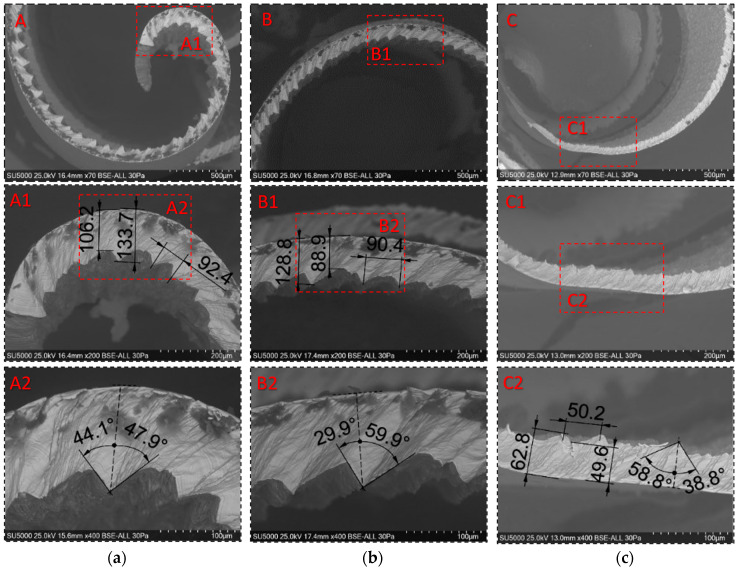
Detailed SEM micrographs of the chip detached during semi-finishing (exp. 7) with different degrees of magnification of the areas specific to the front part of the chip: (**a**) the starting area of the chip (A,A1,A2); (**b**) the middle area of the chip (B,B1,B2); (**c**) the chip end zone (C,C1,C2).

**Figure 17:**
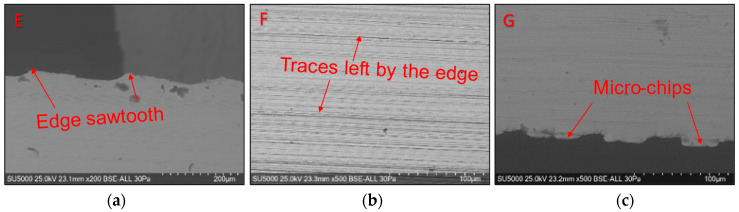
Detailed SEM micrographs of the chip detached during roughing (exp. 2) with different degrees of magnification of the areas specific to the outer side of the chip: (**a**) the free area of the chip (E); (**b**) the middle area of the chip (F); (**c**) the area at the base of the chip (tip of the tool) (G).

**Figure 18:**
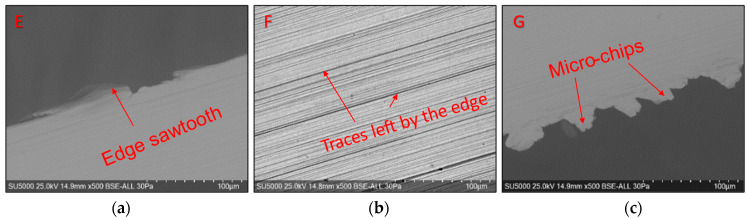
Detailed SEM micrographs of the chip detached during semi-finishing (exp. 7) with different degrees of magnification of the areas specific to the outer side of the chip: (**a**) the free area of the chip (E); (**b**) the middle area of the chip (F); (**c**) the area at the base of the chip (tip of the tool) (G).

**Figure 19:**
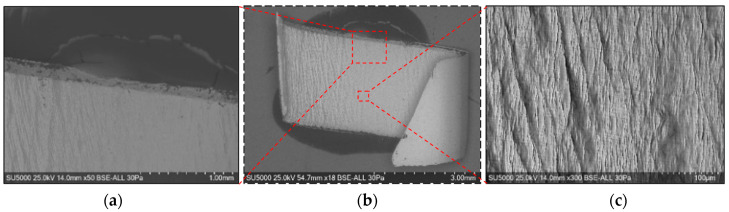
SEM micrographs of the inner area of the chip detached during roughing (exp. 2): (**a**) free area of the chip; (**b**) general view of the area in the middle of the chip; (**c**) detail of the area in the middle of the chip.

**Figure 20:**
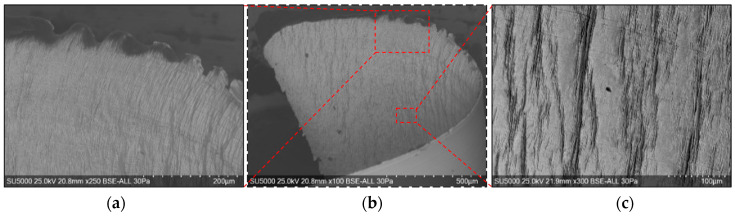
SEM micrographs of the inner area of the chip detached during semi-finishing (exp. 7): (**a**) free area of the chip; (**b**) general view of the area in the middle of the chip; (**c**) detail of the area in the middle of the chip.

Global and detailed SEM micrographs were made for the chips from the roughing (exp. 2) and semi-finishing (exp. 7) experiments. General micrographs, front and side, with the marking of the detailed areas, A, B, and C in front view, respectively, and E, F, and G in side view, are presented in Figure 13 and Figure 14. Details of areas specific to the length of the chip, observed from the front at different degrees of magnification, are presented in the micrographs in Figure 15 and Figure 16. Micrographs with areas specific to the width of the chip, made in the middle area of the length of the chip on its exterior, are presented in Figure 17 and Figure 18. Micrographs of the inside of the chip, made in the middle area of the chip’s length, are presented in Figure 19 and Figure 20.

**Figure 21:**
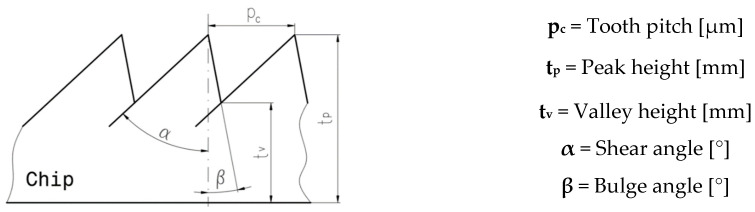
Definition of chip segmentation parameters.

Analysis of the detailed micrographs of the chips in areas specific to their length, as shown in Figure 15 and Figure 16, highlights the fact that the detached chips are serrated (segmented), being determined by stepwise plastic deformation of the material, with a relatively strong connection between the groups of constituent elements [4]. It is observed that these segments have different sizes along the length of the chip, in the specific observed areas A, B, and C, but also from one experiment to another, determined by the different cutting conditions. That is why an analysis of the values of the following chip segmentation parameters was carried out: tooth pitch—p_c_, peak height—t_p_, valley height—t_v_, shear angle—α, and bulge angle—β, as shown in Figure 21, for each of these areas.

Figure 17 and Figure 18 show details of the smooth surface of the chip, which is in contact with the rake face of the tool until the moment the chip curls, when the contact disappears. This surface’s smooth and shiny appearance is the combined effect of high contact pressures, frictional forces, and high temperatures, according to the studies in [11]. For the chips from both experiments, the serrated nature of their upper part is highlighted in image E, as well as the way the chip material is pushed: in the radial direction and the longitudinal direction due to the inclined position of the insert in the milling body. The prominent streaks in the F images represent the marks left by the irregularities on the edge of the cutting insert, as well as by the microchips detached in the formation of the chip. In image G, which shows the lower part of the chip, detachments from the chip material and the formation of micro-chips are observed, which are deformed in several directions by the connecting radius from the tip of the cutting insert, as also shown by Liu D et al. [57].

For the analysis of the internal surfaces of the chips, they were cut in the middle area of the length. The images presented in Figure 19 and Figure 20 reveal the existence of some micro-cracks on the inner surface of the chips, a fact due to the compaction of the material in shear planes, as Wang R et al. also mentioned [5].

**Figure 22:**
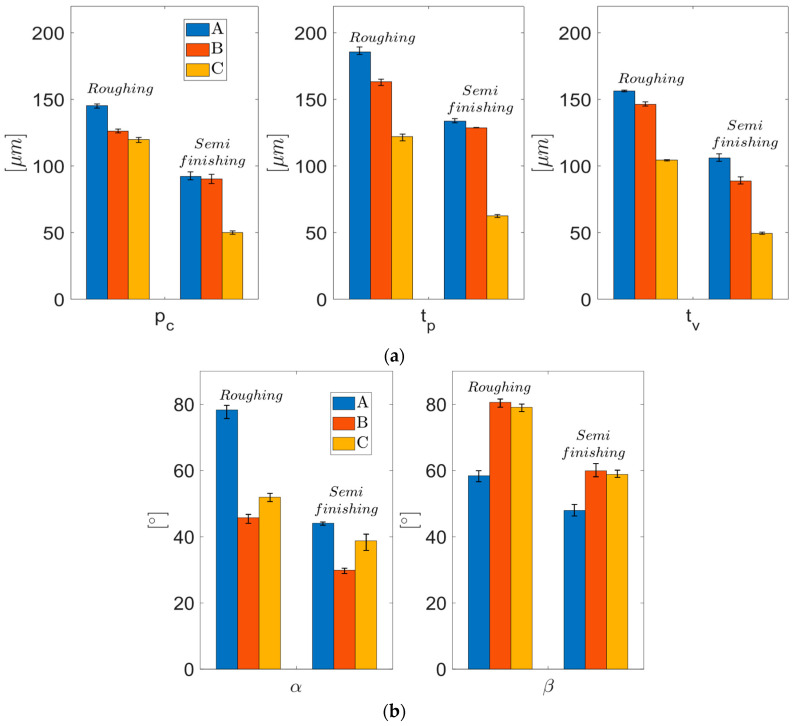
The evolution of the chip segmentation parameters for the two analyzed experiments in specific areas of the chip (the starting area of the chip—A, the middle area of the chip—B, and the chip end zone—C): (**a**) dimensional parameters and (**b**) angular parameters.

Table 4 presents the average values of the chip segmentation parameters, resulting from the measurement in at least three adjacent areas for their statistical analysis, and Figure 22 represents these values graphically.

The analysis highlights the following aspects:

- all the values of the five parameters analyzed are higher for chips detached during roughing than those detached during semi-finishing, a fact explained by the very different intensities of the cutting regimes used in these experiments;
- all the values of the dimensional parameters of chip segmentation have a decreasing evolution, from the starting area of the chip towards the end area of the chip, a fact determined by the decrease in the thickness of the chip, both the uncut and the detached one, as can be seen from Figure 14 and Figure 20;
- the values of the angular parameters of chip segmentation have a different evolution along the chip: while the shear angle α has high values in the starting zone of the chip (A), it decreases in its middle zone (B) and increases again towards the end zone of the chip (C), the bulge angle β has an inverse evolution, with lower values at the beginning of the chip, maximum values in the middle zone, and lower values again in the end zone of the chip. This evolution of the sizes of the two angles is correlated with the evolution of the cutting forces during chip detachment, as presented and analyzed in the previous section: high values of the cutting force during the formation of a chip are associated with a small shear angle and a large camber angle;
- the shear angle α has values below 45 degrees in the case of semi-finishing chips, which means that the chip formation mechanism is shear deformation. In comparison, in the case of roughing, the values of the shear angle α above 45 degrees show that chip deformation is not only a pure shear deformation [11].

For the quantitative analysis of the chip segmentation, the indicator of the degree of chip segmentation, G [12], was also evaluated:

(4) $$G=\frac{t_{p\;}-t_{v}}{t_{p}}$$

**Figure 23:**
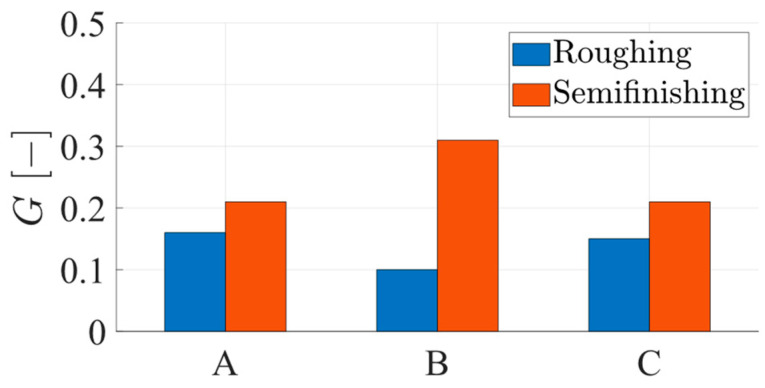
The evolution of the degree of chip segmentation, G, along the chip.

The graphic representation of the values of this indicator along the chip, as shown in Figure 23, highlights the following aspects:

- the values of the degree of chip segmentation, G, are higher in the case of semi-finishing chips, where it was shown that the mechanism of chip formation is shear deformation;
- the degree of segmentation of the chips has relatively close values in the area of the beginning, respectively, of the end of the chip, but the values in the median zone of the chip length are different from these: lower in the case of roughing chips results in a more intense cutting operation, respectively, higher in the case of semi-finishing chips results at a less intense cutting operation.

The analysis presented in this section of the paper also highlighted that, from the point of view of the morphology of the detached chips, their characteristics, both macroscopic and microscopic, are influenced by the values of the cutting parameters, with major differences between those obtained at semi-finishing processing and those resulting from roughing processing.

### 3.5. Influence of the Cutting Parameters on the Microscopic Dimensions of the Chips

Building upon the findings of the previous sections, particularly the correlation between the dimensions of the detached chip and the intensity of the cutting conditions, this section presents a meticulously crafted mathematical model. This model precisely maps the dependence of the dimensions of the detached chips, d_c_ and h_c_, as response variables, on the cutting parameters, a_p_, f_z_, and v_c_, as input parameters, in the form of process functions.

**Figure 24:**
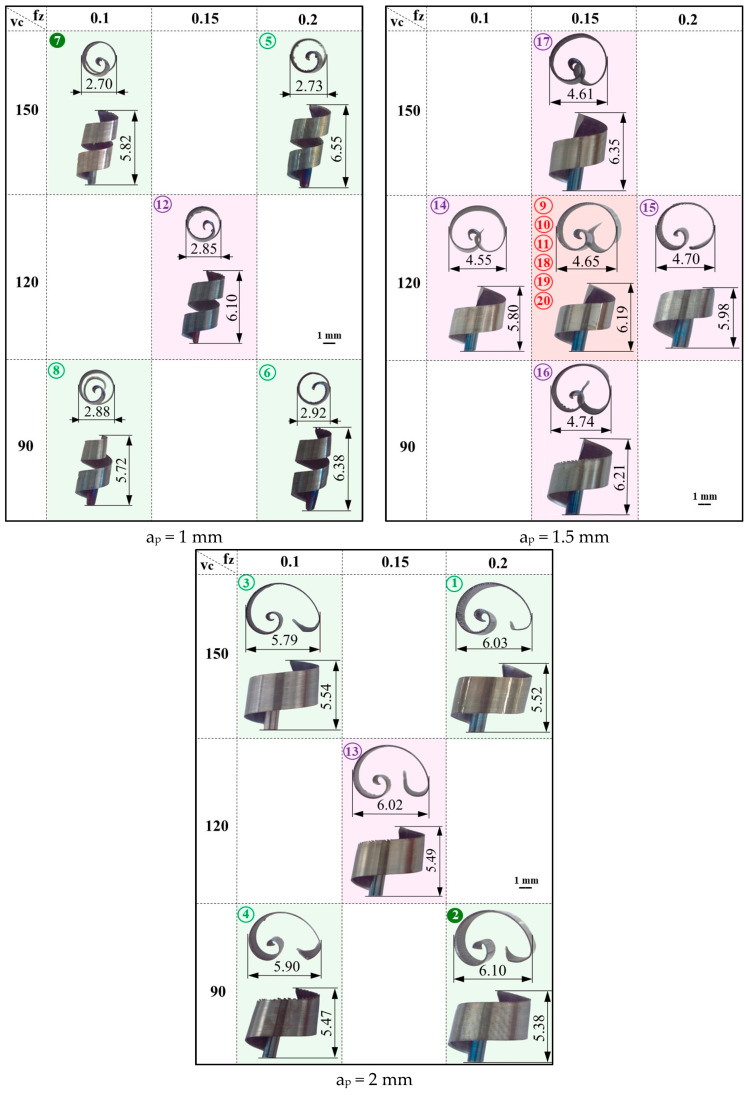
Chip sizes for different cutting parameters (the numbers in circles and the background colors of the images are associated with those of the experiments, marked in Table 2).

The dimensions of the detached chips, a result of a systematic approach involving 20 experiments with the central composite design, were meticulously determined based on optical micrographs. These results are presented in Figure 24, providing a clear visual representation of the data.

The analysis of these dependencies starts from the following premises: (1) the volume of the uncut chip is determined by the parameter’s width—l_c_, thickness—t_c_, and length—L_c_, as shown in Figure 5a, and is constant for a set of values of the parameters of the cutting conditions; (2) the uncut chip length—L_c_ is constant in the experimental results because the chip width ae was constant; (3) the volume of the detached chip is equal to the volume of the uncut chip [44]. Therefore, the uncut chip volume remains constant in experiments where only the cutting speed is varied. Because L_c_ is constant in the experiments carried out, there will be an inversely proportional relationship between the response variable “maximum chip height—h_c_” and the response variable “maximum chip curling diameter—d_c_”.

Because the experiments were carried out based on the central composite design, the response surface methodology (RSM) was used to determine the process functions [58]. The mathematical model that expresses the dependence of the sizes of detached chips on the parameters of the cutting regime is of the second-order polynomial type:

*f*(*a_p_*,*f_z_*,*v_c_*) = b_0_ + b_1_*a_p_* + b_2_*f_z_* + b_3_*v_c_* + b_4_*a_p_*^2^ + b_5_*f_z_*^2^ + b_6_*v_c_*^2^ + b_7_*a_p_f_z_* + b_8_*a_p_v_c_* + b_9_*f_z_v_c_* (5)

where f is, as the case may be, d_c_ or h_c_, and b0, … b9 are the coefficients of the function, which are determined by multivariable regression analysis.

For this purpose, the MINITAB 20 Statistical software was used to carry out the analysis of variance (ANOVA). ANOVA was used to statistically evaluate, for a confidence level of 95% (alpha = 0.05), the influence of the input parameters and their interactions on the response variable. This analysis was carried out on the adequacy of the model and the significance of the mathematical model terms by analyzing the *p*-values (null hypothesis testing) and F-values (Fisher distribution).

The analyzed mathematical model must be adequate (to correctly represent the experimental data), and the *p*-value for lack-of-fit must be greater than 0.05.

For the term of the mathematical model to be significant, the value of *p* must be less than 0.05. The smaller the value of *p*, the greater the influence of the term. When small *p* values are difficult to determine (due to software limitations in displaying very small values), the value of F is also analyzed. The largest value of F will lead to the smallest value of *p* [59].

Suppose one or more terms of an appropriate mathematical model is/is not/are/are not significant. In that case, a new model can be determined and analyzed, derived from the initial one, by eliminating the insignificant term/terms. The new mathematical model is better than the initial one if it has lower values for standard deviation (SD), the prediction error sum of squares (PRESS), Corrected Akaike’s Information Criterion (AICc), and Bayesian Information Criterion (BIC), and the value R-sq(pred) is higher than that of the original model [60,61]. Additionally, the closer the values for the coefficients of determination R-sq, R-sq(adj), and R-sq(pred) are to 100%, the better the model describes the experimental data.

The ANOVA results for the process function “maximum chip curling diameter” are presented in Table 5 for the initial model. The percentage contribution ratio (PCR) shows the proportion of the total variation of each factor, expressed as a percentage of the total sum of squares. The PCR reflects the percentage contribution of each factor to the total response variation. It is found that the model is adequate (*p*-value for lack-of-fit = 0.523 > 0.05), the linear component has a very high weight (over 98%), the terms v_c_ ∙ v_c_ and f_z_ ∙ v_c_ are insignificant (they have *p*-values > 0.05), and the other terms have a very small contribution (percentage contribution ratio—PCR). We adjusted the model to reduce the number of terms but have a lack-of-fit value greater than 0.05. As a result, we have a model 5DOF that adequately fits the data, maintaining a balance between model complexity and accuracy. Table 6 presents the synthesis of the results of the ANOVA analysis for the two mathematical models, the initial one with 9 degrees of freedom and the final model with 5 degrees of freedom. The second mathematical model (6) is adopted because it presents better result indicators than those of the initial model.

(6) $$d_{c}=-1.902+5.958a_{p}-1.455f_{z}-0.002267v_{c}-1.028a_{p}^{2}+1.85a_{p}f_{z}$$

**Figure 25:**
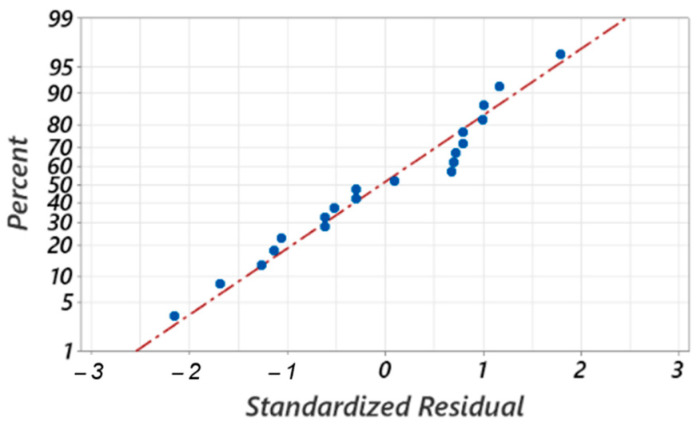
Standardized residual plots for the maximum curling diameter of the chip.

Figure 25 shows the standardized residuals of the second model (adopted model) represented as randomly distributed about the regression line and close to it, which proves that the errors are normally distributed and that the model adequately represents the experimental data.

**Figure 26:**
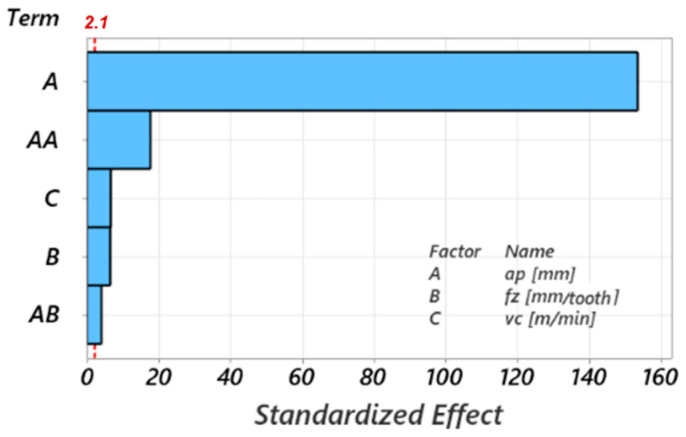
Pareto chart of standardized effects for the maximum curling diameter of the chip.

Figure 26 presents the Pareto chart of standardized effects, showing the influence of the terms of the adopted mathematical model on the response variable. The Pareto chart of standardized effects is used to identify the significant factors in an experiment by ordering the effects according to their standardized size. The effects are calculated by dividing the regression coefficients by the standard deviation associated with the coefficients. This allows a direct comparison of the relative importance of each factor. The cutting depth has the greatest influence on the chip diameter, with a very high weight of over 98%.

**Figure 27:**
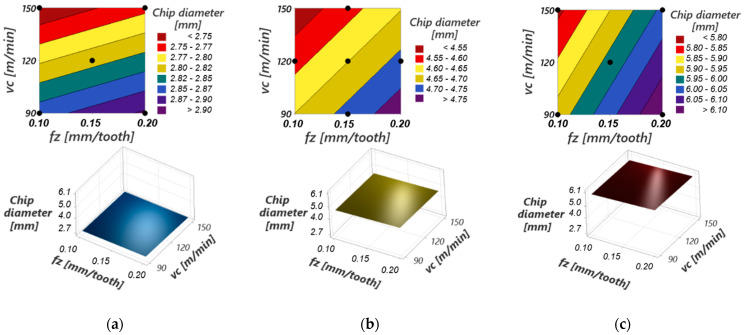
3D interaction plots of the maximum curling diameter of the chip versus the cutting speed and feed per tooth for: (**a**) cutting depth =1 mm; (**b**) cutting depth = 1.5 mm; (**c**) cutting depth = 2 mm.

Figure 27 presents graphic representations of the response surfaces for the process function “maximum chip curling diameter” for different values of cutting speed and feed per tooth at constant cutting depths. The response surfaces have a curvilinear profile due to the quadratic terms in the model (especially ap) and the interactions between the input variables (ap with fz, in particular). However, this curvature of the response surface is not pronounced because the influence of the mentioned terms is not important within the model.

The maximum curling diameter of the detached chip increases with the increase in the cutting depth, thus having the greatest influence within the model. This is determined by the fact that as the cutting depth increases, the width of the uncut chip increases, increasing the resistance of the material in the chip to curling [7].

At a constant value of the cutting depth, the maximum curling diameter of the detached chip increases with increasing feed per tooth because of the uncut chip thickness; thus, its resistance to curling increases. According to the results of the ANOVA analysis, Table 5, and Figure 26, this influence is much less important than that produced by increasing the cutting depth.

At a constant value of the cutting depth, the maximum curling diameter of the detached chip decreases with the increase in the cutting speed, an evolution explained by the temperature increase at the tool-chip interface [62]. Thus, a large temperature difference is created between the contact area of the chip with the tool and chip-free surfaces, causing it to curl. This chip curling effect at higher temperatures is also favored by the decrease in the deformation resistance of the material in the detached chip [63].

The smallest value of the maximum curling diameter of the detached chip is obtained at minimum values of the cutting depth and feed per tooth and maximum values of the cutting speed (a_p_ = 1 mm, f_z_ = 0.1 mm/tooth, v_c_ = 150 m/min), and the highest value is obtained at maximum values of the cutting depth and feed per tooth and minimum values of the cutting speed (a_p_ = 2 mm, f_z_ = 0.2 mm/tooth, v_c_ = 90 m/min).

The ANOVA results for the process function “maximum chip height” are presented in Table 7 for the initial model. It is found that the model is adequate (*p*-value for lack-of-fit = 0.053 > 0.05), its linear component is the majority (over 53%)—but not as important as in the “chip curling diameter” process function, and the terms a_p_ ∙ v_c_ and f_z_ ∙ v_c_ are insignificant (have *p*-value > 0.05).

Therefore, a second model was analyzed, derived from the initial one, by eliminating the previously mentioned terms. Table 8 presents the synthesis of the ANOVA analysis results for the two mathematical models: the initial one with 9 degrees of freedom and the final model with 7 degrees of freedom. The second mathematical model (7) is adopted because it presents better result indicators than those of the initial model.

(7) $$h_{c}=2.535+4.309a_{p}+40.95f_{z}-0.04242v_{c}-1.273a_{p}^{2}-89.3f_{z}^{2}+0.000185v_{c}^{2}+7.5a_{p}f_{z}$$

**Figure 28:**
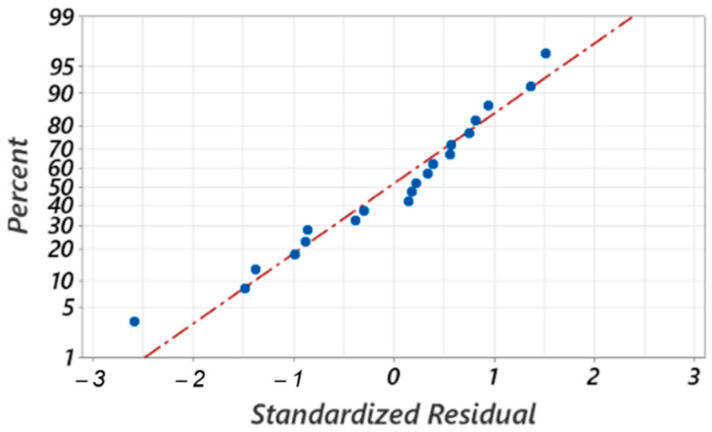
Standardized residual plots for the maximum height of the chip.

Figure 28 shows the second model’s standardized residuals. They are randomly distributed about the regression line and very close to it, indicating that the errors are normally distributed and that the model adequately represents the experimental data.

**Figure 29:**
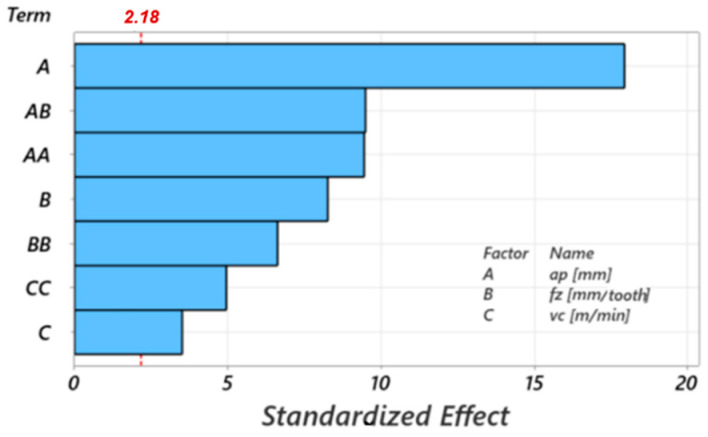
Pareto chart of standardized effects for the maximum height of the chip.

The Pareto chart of standardized effects in Figure 29 shows the influence of the terms of the adopted mathematical model on the response variable “maximum chip height.” In this process function, the cutting depth has the greatest influence within the model, but the other terms, especially the feed per tooth, have greater influences.

The maximum height of the detached chip decreases with the increase in the cutting depth, thus having the greatest influence within the model. This evolution of the chip height is determined by the fact that as the cutting depth increases, the diameter of the detached chip increases (see the previous explanations) and that between the response variable “maximum chip height—h_c_” and the response variable “maximum curling diameter of the chip—dc”, there is an inverse proportional relationship (see the premises mentioned at the beginning of the section).

**Figure 30:**
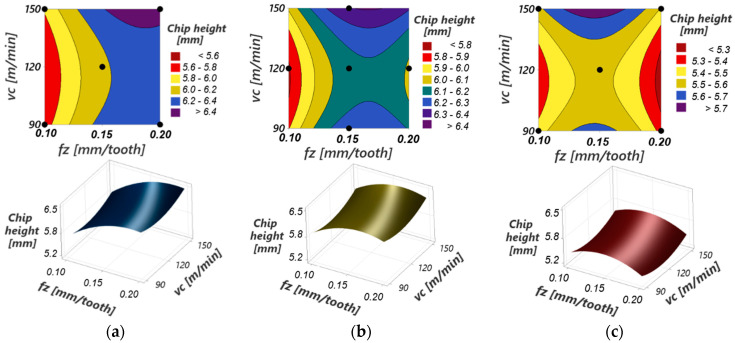
3D interaction plots of the maximum height of the chip versus cutting speed and feed per tooth for: (**a**) cutting depth =1 mm; (**b**) cutting depth = 1.5 mm; (**c**) cutting depth = 2 mm.

Figure 30 presents graphic representations of the response surfaces for the “maximum chip height” process function for different values of cutting speed and feed per tooth at constant cutting depths.

The response surfaces have a pronounced curvilinear profile in the form of a “saddle”, with a convex shape in the h_c_ –f_z_ plane and a concave shape in the h_c_–v_c_ plane. These shapes are due to the influences of the quadratic terms in the model (especially ap) and the interactions between the input parameters a_p_ and f_z_ (the interactions between the other input variables are insignificant), which are important. Thus, the following dependencies of the input variables on the response variable “maximum chip height” are highlighted:

- At constant values of the cutting depth and cutting speed, the maximum height of the detached chip has a maximum value for a feed per tooth f_z_ between the experimental limits (0.1 and 0.2 mm/tooth). The value of this maximum decreases with the increase in the cutting depth.
- At constant values of the cutting depth and feed per tooth, the maximum height of the detached chip has a minimum value for a cutting speed v_c_ between the experimental limits (90 and 150 m/min).

The lowest value of the maximum height of the detached chip is obtained at maximum values of the cutting depth and feed per tooth and average values of the cutting speed (a_p_ = 2 mm, f_z_ = 0.2 mm/tooth, v_c_ = 120 m/min), and the highest value is obtained at minimum values of the cutting depth and maximum values of the feed per tooth and cutting speed (a_p_ = 1 mm, f_z_ = 0.2 mm/tooth, v_c_ = 150 m/min).

From the point of view of chip removal from the cutting area, both its diameter and its height should be small. As this cannot be achieved simultaneously, the chip diameter should be small so that the cutting effort is small and the contact area with the tool tooth is smaller, which will decrease the tool wear intensity. However, it must be considered that cutting with small cutting depths significantly reduces machining productivity.

## 4. Conclusions

The research conducted in this study has brought to light several important aspects for understanding the mechanisms and phenomena related to the formation and removal of chips, as well as for selecting the appropriate values of the parameters for the chipping process.

The comprehensive analysis of chip and process temperatures, cutting forces, and chip morphology for roughing and semi-finishing machining led to valuable conclusions. The most important of these are the following.

- The two machining methods being investigated show similarities in the evolution of temperatures and cutting forces during the machining, as well as in the variation of dimensional and angular parameters of chip segmentation. These similarities are due to the similar mechanisms of chip formation and shear deformation in both processes.
- The two machining methods differ in the levels of temperature, cutting forces, and dimensional and angular parameters of chip segmentation. In roughing machining, these differences are higher due to the larger dimensions of the uncut chip, which significantly impacts the phenomena occurring at the workpiece-tool-chip interfaces. This leads to a sharp increase in both mechanical and thermal processes during material removal.

Regarding the impact of the cutting parameters on the overall dimensions of the chips, the main findings are:

- This study has shown that it is possible to use regression functions to mathematically model the dependence of the separated chips’ size on the cutting process parameters. The functions identified are second-order polynomials and are considered suitable with high coefficients of determination at a confidence level of 95% (alpha = 0.05).
- To achieve small-sized chips, it is important to set the cutting parameters in the following order: cutting depth, feed per tooth, and cutting speed, with the lowest possible values for the first two while maintaining productivity during the machining process.

This study can be further developed by analyzing the integrity of machined surfaces and tool wear, modeling and simulating the process to investigate the chip formation process, and identifying the optimal cutting conditions for different criteria.

## Figures and Tables

**Table 1:** Chemical composition of 55NiCrMoV7 steel.

Chemical Element	C	Fe	Si	Ni	Cr	Mn	Mo	V	S	*p*
Effective values (wt%)	0.56 (ISO)	93.68	1.231	1.717	1.174	0.963	0.3	0.103	0.2	0.004
Measurement error (%)	-	0.07	0.01	0.008	0.004	0.005	0.001	0.002	-	-

**Table 2:** Measured mechanical properties of 55NiCrMoV7 steel.

Hardness [HV]	Ultimate Tensile Stress [MPa]	Relative Elongation [%]
477.4 ± 3	1371.7	7.1

**Table 3:** Central composite design.

Exp. nb.	Normalized Independent Variables			Physical Independent Variables		
	a_p_ [mm]	f_z_ [mm/Tooth]	v_c_ [m/min]	a_p_ [mm]	f_z_ [mm/Tooth]	v_c_ [m/min]
**1.**	1	1	1	*2*	*0.2*	*150*
**2.**	1	1	−1	*2*	*0.2*	*90*
**3.**	1	−1	1	*2*	*0.1*	*150*
**4.**	1	−1	−1	*2*	*0.1*	*90*
**5.**	−1	1	1	*1*	*0.2*	*150*
**6.**	−1	1	−1	*1*	*0.2*	*90*
**7.**	−1	−1	1	*1*	*0.1*	*150*
**8.**	−1	−1	−1	*1*	*0.1*	*90*
**9.**	0	0	0	*1.5*	*0.15*	*120*
**10.**	0	0	0	*1.5*	*0.15*	*120*
**11.**	0	0	0	*1.5*	*0.15*	*120*
**12.**	−1	0	0	*1*	*0.15*	*120*
**13.**	1	0	0	*2*	*0.15*	*120*
**14.**	0	−1	0	*1.5*	*0.1*	*120*
**15.**	0	1	0	*1.5*	*0.2*	*120*
**16.**	0	0	−1	*1.5*	*0.15*	*90*
**17.**	0	0	1	*1.5*	*0.15*	*150*
**18.**	0	0	0	*1.5*	*0.15*	*120*
**19.**	0	0	0	*1.5*	*0.15*	*120*
**20.**	0	0	0	*1.5*	*0.15*	*120*

**Table 4:** Average values of chip segmentation parameters for the two analyzed experiments.

Chip Parameter’s		UM	Roughing			Semi-Finishing		
			A	B	C	A	B	C
* Φ	positioning angle of the tooltip	°	12	24	46	12	24	46
* L_c_	uncut chip length	mm	5	10	20	5	10	20
* t_c_	uncut chip thickness	µm	167	157	119	83	78	60
t_p_	chip peak height	µm	185.7	163.3	122.2	133.7	128.8	62.8
t_v_	chip valley height	µm	156.5	146.4	104.4	106.2	88.9	49.6
p_c_	tooth pitch between shear planes	µm	145.4	126.3	119.9	92.4	90.4	50.2
α	shear angle	°	78.3	45.7	51.9	44.1	29.9	38.8
β	bulge angle	°	58.4	80.6	79.1	47.9	59.9	58.8

* The values for Φ, L_c_, and t_c_ are approximate to indicate the position of areas A, B, and C.

**Table 5:** ANOVA for the process function “maximum chip curling diameter”—initial model.

Source	DOF	Seq SS	PCR	Adj SS	Adj MS	F-Value	*p*-Value
**Model**	9	25.2852	99.98%	25.2852	2.8095	6320.20	0.000
***Linear***	3	24.9276	98.57%	24.9276	8.3092	18,692.38	0.000
*a_p_*	1	24.8378	98.21%	24.8378	24.8378	55,875.12	0.000
*f_z_*	1	0.0436	0.17%	0.0436	0.0436	97.99	0.000
*v_c_*	1	0.0462	0.18%	0.0462	0.0462	104.02	0.000
***Square***	3	0.3360	1.33%	0.3360	0.1120	251.92	0.000
*a_p_ ∙ a_p_*	1	0.3302	1.31%	0.1507	0.1507	339.01	0.000
*f_z_ ∙ f_z_*	1	0.0056	0.02%	0.0053	0.0053	12.03	0.006
*v_c_ ∙ v_c_*	1	0.0001	0.00%	0.0001	0.0001	0.22	0.652
***2-Way Interaction***	3	0.0217	0.09%	0.0217	0.0072	16.30	0.000
*a_p_ ∙ f_z_*	1	0.0171	0.07%	0.0171	0.0171	38.50	0.000
*a_p_ ∙ v_c_*	1	0.0045	0.02%	0.0045	0.0045	10.15	0.010
*f_z_ ∙ v_c_*	1	0.0001	0.00%	0.0001	0.0001	0.25	0.626
Error	10	0.0044	0.02%	0.0044	0.0004		
***Lack-of-Fit***	5	0.0022	0.01%	0.0022	0.0004	0.95	0.523
**Pure Error**	5	0.0023	0.01%	0.0023	0.0005		
**Total**	19	25.2897	100.00%				

**Table 6:** Synthesis with the results of the ANOVA analysis, the “maximum chip curling diameter” function.

Model	*SD*	*R-sq*	*R-sq(adj)*	*PRESS*	*R-sq(pred)*	*AICc*	*BIC*
**9DOF** (Iniţial)	0.021083	99.98%	99.97%	0.019496	99.92%	−56.48	−78.52
**5DOF** (Final)	0.032489	99.94%	99.92%	0.039407	99.84%	−64.12	−66.48

**Table 7:** ANOVA for process function “maximum chip height”—initial model.

Source	DOF	Seq SS	PCR	Adj SS	Adj MS	F-Value	*p*-Value
**Model**	9	2.31925	98.53%	2.31925	0.25769	74.54	0.000
***Linear***	3	1.25649	53.38%	1.25649	0.41883	121.15	0.000
*a_p_*	1	1.00489	42.69%	1.00489	1.00489	290.68	0.000
*f_z_*	1	0.21316	9.06%	0.21316	0.21316	61.66	0.000
*v_c_*	1	0.03844	1.63%	0.03844	0.03844	11.12	0.008
***Square***	3	0.77861	33.08%	0.77861	0.25954	75.07	0.000
*a_p_ ∙ a_p_*	1	0.61952	26.32%	0.27841	0.27841	80.53	0.000
*f_z_ ∙ f_z_*	1	0.08256	3.51%	0.13698	0.13698	39.62	0.000
*v_c_ ∙ v_c_*	1	0.07653	3.25%	0.07653	0.07653	22.14	0.001
***Two-Way Interaction***	3	0.28415	12.07%	0.28415	0.09472	27.40	0.000
*a_p_ ∙ f_z_*	1	0.28125	11.95%	0.28125	0.28125	81.35	0.000
*a_p_ ∙ v_c_*	1	0.00045	0.02%	0.00045	0.00045	0.13	0.726
*f_z_ ∙ v_c_*	1	0.00245	0.10%	0.00245	0.00245	0.71	0.420
**Error**	10	0.03457	1.47%	0.03457	0.00346		
***Lack-of-Fit***	5	0.02869	1.22%	0.02869	0.00574	4.88	0.053
**Pure Error**	5	0.00588	0.25%	0.00588	0.00118		
**Total**	19	2.35382	100.00%				

**Table 8:** Synthesis with the results of the ANOVA analysis, the “maximum chip height” function.

Model	*SD*	*R-sq*	*R-sq(adj)*	*PRESS*	*R-sq(pred)*	*AICc*	*BIC*
**9DOF** (Initial)	0.058797	98.53%	97.21%	0.157325	93.32%	−15.45	−37.50
**7DOF** (Final)	0.055880	98.41%	97.48%	0.119535	94.92%	−32.84	−41.88

## Data Availability

The corresponding author will provide the data used in this work upon reasonable request.

## Nomenclature

F_X_	Force measured in the X-axis direction	p_c_	Tooth pitch between shear planes
F_Y_	Force measured in the Y-axis direction	t_p_	Chip peak height
F_Z_	Force measured in the Z-axis direction	t_v_	Chip valley height
F_t_	Tangential cutting force	α	Shear angle
F_r_	Radial cutting force	β	Bulge angle
F_p_	Inward force	G	Degree of segmentation
F_c_	Cutting force	ANOVA	Analysis of variance
Φ	Positioning angle of the tooltip	RSM	Response surface methodology
Φ_a_	Active machining angle	CCD	Central composite design
a_p_	Cutting depth	DOF	Degrees of freedom
f_z_	Feed per tooth	SeqSS	Sequential sum of squares
v_c_	Cutting speed	PCR	Percentage Contribution Ratio
a_e_	Cutting width	AdjSS	Adjusted sum of squares
l_f_	Machining length	AdjMS	Adjusted mean squares
D_c_	Tool diameter	F-Value	Fisher distribution
z_c_	Number of tool teeth	*p*-Value	Null-hypothesis significance testing
K	Entering angle	SD	Standard deviation
d_c_	Maximum chip curling diameter	R-sq	Coefficient of determination R^2^
h_c_	Maximum chip height	R-sq (adj)	Adjusted R^2^
l_c_	Uncut chip width	PRESS	Predicted residual error sum of squares
t_c_	Uncut chip thickness	R-sq (pred)	Predicted R^2^
L_c_	Uncut chip length	AICc	Corrected Akaike’s Information Criterion
		BIC	Bayesian Information Criterion

## References

[1] Patel G.C.M., Chate G.R., Parappagoudar M.B., Gupta K. (2020). Machining of Hard Materials: A Comprehensive Approach to Experimentation, Modeling and Optimization.

[2] Davim J.P. (2011). Machining of Hard Materials.

[3] Wang G., Zhou X., Wu X., Ma J. (2019). Failure and Control of PCBN Tools in the Process of Milling Hardened Steel. Metals.

[4] Singh B.K., Roy H., Mondal B., Roy S.S., Mandal N. (2019). Measurement of chip morphology and multi criteria optimization of turning parameters for machining of AISI 4340 steel using Y-ZTA cutting insert. Measurement.

[5] Wang R., Wang X., Yan P., Zhou T., Jiao L., Teng L., Zhao B. (2023). The effects of cryogenic cooling on tool wear and chip morphology in turning of tantalum-tungsten alloys Ta-2.5W. J. Manuf. Process..

[6] Elbah M., Fnides B., Laouici H., Yallese A. (2021). Modélisation et optimisation des conditions de coupe en tournage dur par la technique de Taguchi en utilisant la MSR. UPB Sci. Bull. Ser. D.

[7] Cui X., Zhao J. (2015). Effects of cutting parameters on chip morphology and tool wear in high-speed face milling of hardened steel. Proc. Inst. Mech. Eng. Part B J. Eng. Manuf..

[8] Li S., Zheng G., Ding F., Zhang J., Cheng X., Cui E. (2024). Influence of clean cooling medium on the hard drilling performance and machined surface integrity of 42CrMo steel. Mater. Today Commun..

[9] Lazoglu I., Boz Y., Erdim H. (2011). Five-axis milling mechanics for complex free form surfaces. CIRP Ann..

[10] Yao Z., Shen J., Wu M., Zhang D., Luo M. (2023). Position-dependent milling process monitoring and surface roughness prediction for complex thin-walled blade component. Mech. Syst. Signal Process..

[11] Zhang S., Guo Y.B. (2009). An experimental and analytical analysis on chip morphology, phase transformation, oxidation, and their relationships in finish hard milling. Int. J. Mach. Tools Manuf..

[12] Liu H., Zhang J., Jiang Y., He Y., Xu X., Zhao W. (2016). Investigation on Morphological Evolution of Chips for Ti6Al4V Alloys with the Increasing Milling Speed. Procedia CIRP.

[13] Hariprasad B., Selvakumar S.J., Samuel R.D. (2022). Effect of cutting edge radius on end milling Ti–6Al–4V under minimum quantity cooling lubrication—Chip morphology and surface integrity study. Wear.

[14] Chen X., Tang J., Ding H., Liu A. (2021). Experimental study on the evolution of chip morphology, chip formation, and surface topography with cutting parameters, and their relationships in dry milling of cast aluminum alloy with PCD inserter. J. Mech. Sci. Technol..

[15] Alipanahi A., Mahboubkhah M., Barari A. (2022). Cross-sensitivity control in a novel four-component milling dynamometer for simultaneous measurement of tri-axial forces and torque. Measurement.

[16] Gomez M.F., Schmitz T.L. (2019). Displacement-based dynamometer for milling force measurement. Procedia Manuf..

[17] Gomez M., Schmitz T. (2020). Low-cost, constrained-motion dynamometer for milling force measurement. Manuf. Lett..

[18] Uddin M.S., Songyi D. (2016). On the design and analysis of an octagonal–ellipse ring based cutting force measuring transducer. Measurement.

[19] Zhou C., Guo K., Zhao Y., Zan Z., Sun J. (2020). Development and testing of a wireless rotating triaxial vibration measuring tool holder system for milling process. Measurement.

[20] Rizal M., Ghani J.A., Nuawi M.Z., Haron C.H.C. (2018). An embedded multi-sensor system on the rotating dynamometer for real-time condition monitoring in milling. Int. J. Adv. Manuf. Technol..

[21] Qin Y., Zhao Y., Li Y., Zhao Y., Wang P. (2017). A novel dynamometer for monitoring milling process. Int. J. Adv. Manuf. Technol..

[22] Xu J., Shen J., Li L., Guo G., Zhu X., Meng Y., Chen M. (2023). Milling machinability analysis of GW63K rare-earth magnesium alloys based on the concept of clean cutting. J. Mater. Res. Technol..

[23] Fan G., Zhang J., Zhang G., Xu C., Yi M. (2023). Finite element analysis of Ti1-xAlxN coated tools cutting performance and tool wear during Ti–6Al–4V milling. J. Mater. Res. Technol..

[24] Liu X., Wang W., Jiang R., Xiong Y., Lin K., Li J., Shan C. (2022). Analytical model of workpiece temperature in axial ultrasonic vibration-assisted milling in situ TiB2/7050Al MMCs. Int. J. Adv. Manuf. Technol..

[25] Zhang X., Zhang J., Zhao W. (2016). A new method for cutting force prediction in peripheral milling of complex curved surface. Int. J. Adv. Manuf. Technol..

[26] Cui X., Zhao J., Tian X. (2013). Cutting forces, chip formation, and tool wear in high-speed face milling of AISI H13 steel with CBN tools. Int. J. Adv. Manuf. Technol..

[27] Günay M. (2022). Modeling and multiple optimization in face milling of hardfacing welding applied steel: Force, roughness, power. Proc. Inst. Mech. Eng. Part C J. Mech. Eng. Sci..

[28] Cui X.B., Guo J.X., Wang X.Y. (2015). Cutting Force in High-Speed Face Milling AISI H13 Steel. Key Eng. Mater..

[29] Karaguzel U., Bakkal M., Budak E. (2016). Modeling and Measurement of Cutting Temperatures in Milling. Procedia CIRP.

[30] Bolar G., Adhikari R., Nayak S.N., Joshi S.N. (2022). Assessment of ignition risk in dry helical hole milling of AZ31 magnesium alloy considering the machining temperature and chip morphology. J. Manuf. Process..

[31] Varatharajulu M., Duraiselvam M., Krishna P.G.V., Jagadeesh B. (2023). Tool temperature thermographic study on end milling magnesium AZ31 using carbide tool. Mater. Chem. Phys..

[32] Li Y., Zheng G., Cheng X., Yang X., Xu R., Zhang H. (2019). Cutting Performance Evaluation of the Coated Tools in High-Speed Milling of AISI 4340 Steel. Materials.

[33] John R., Lin R., Jayaraman K., Bhattacharyya D. (2023). Investigation on microstructure characteristics of tool wear and machined surface mechanisms while milling: Kenaf vs glass fiber-reinforced composites. J. Mater. Res. Technol..

[34] Zhou R. (2020). Analytical model of workpiece surface temperature prediction in 4-axis milling process. Int. J. Adv. Manuf. Technol..

[35] Cui X., Guo J., Zhao J., Yan Y. (2015). Chip temperature and its effects on chip morphology, cutting forces, and surface roughness in high-speed face milling of hardened steel. Int. J. Adv. Manuf. Technol..

[36] Cheng Y., Guan R., Lu Z., Xu M., Liu Y. (2018). A study on the milling temperature and tool wear of difficult-to-machine 508III steel. Proc. Inst. Mech. Eng. Part B J. Eng. Manuf..

[37] Niu J., Huang C., Shi Z., Liu H., Tang Z., Su R., Chen Z., Li B., Wang Z., Xu L. (2024). A chip formation mechanism taking into account microstructure evolution during the cutting process: Taking compacted graphite iron machining as an example. Int. J. Mach. Tools Manuf..

[38] Nakayama K., Arai M., Kanda T. (1988). Machining Characteristics of Hard Materials. CIRP Ann..

[39] Niu W., Wang Y., Li X., Guo R. (2023). A Joint Johnson–Cook-TANH Constitutive Law for Modeling Saw-Tooth Chip Formation of Ti-6AL-4V Based on an Improved Smoothed Particle Hydrodynamics Method. Materials.

[40] Carvalho S., Horovistiz A., Davim J.P. (2023). Morphological characterization of chip segmentation in Ti-6Al-7Nb machining: A novel method based on digital image processing. Measurement.

[41] Vipindas K., Mathew J. (2021). Analysis of chip morphology to understand the machining mechanism of micro end milling while machining Ti-6Al-4V. Mater. Today Proc..

[42] Wang B., Liu Z., Huo X., Zhao J. (2018). Influences of Cutting Speed and Material Mechanical Properties on Chip Deformation and Fracture during High-Speed Cutting of Inconel 718. Materials.

[43] Zou Z., He L., Jiang H., Yuan H. (2021). Influence of Microgroove Structure on Cutting Performance and Chip Morphology during the Turning of Superalloy Inconel 718. Materials.

[44] Wagner V., Vissio A., Duc E., Pijolat M. (2016). Relationship between cutting conditions and chips morphology during milling of aluminium Al-2050. Int. J. Adv. Manuf. Technol..

[45] Khan M.A., Imran J.S.H., Khan M., Alruqi M. (2023). Machinability analysis of Ti-6Al-4V under cryogenic condition. J. Mater. Res. Technol..

[46] Bhandarkar L.R., Behera M., Mohanty P.P., Sarangi S.K. (2021). Experimental investigation and multi-objective optimization of process parameters during machining of AISI 52100 using high performance coated tools. Measurement.

[47] Deng Q., Li D., Wang H., Cao P., Wu Y., Wang S. (2023). Study of the noise reduction by optimizing ultra-high speed milling parameters by DoE method. UPB Sci. Bull. Ser. D.

[48] Kumar S.S., Bara A., Bhaskar P., Sai K.K., Rajiv L.S., Singh S.L. (2021). Optimization of process parameters based on RSM and GRA method for machining of Inconel-600 by electric discharge machining. Mater. Today Proc..

[49] Chakraborty S., Mitra S., Bose D. (2021). An investigation on dimensional accuracy and surface topography in powder mixed WEDM using RSM and GRA-PCA. Mater. Today Proc..

[50] Koli Y., Yuvaraj N., Aravindan S.V. (2021). Multi-response mathematical model for optimization of process parameters in CMT welding of dissimilar thickness AA6061-T6 and AA6082-T6 alloys using RSM-GRA coupled with PCA. Adv. Ind. Manuf. Eng..

[51] (2018). Metallic Materials- Vickers hardness test—Part 1: Teste Method.

[52] (2018). Metallic Materials- Vickers hardness test—Part 2: Verification and Calibration of Testing Machines.

[53] (2009). Metallic Materials—Tensile Testing—Part 1: Method of Test at Room Temperature.

[54] Smith G.T. (2008). Cutting Tool Technology.

[55] Wang D., Yin L., Hänel A., Teicher U., Penter L., Seidel A., Harst S., Ihlenfeldt S. (2023). Cutting performance of binderless nano-polycrystalline cBN and PcBN milling tools for high-speed milling of hardened steel. Ceram. Int..

[56] Yıldırım Ç.V., Kıvak T., Sarıkaya M., Şirin Ş. (2020). Evaluation of tool wear, surface roughness/topography and chip morphology when machining of Ni-based alloy 625 under MQL, cryogenic cooling and CryoMQL. J. Mater. Res. Technol..

[57] Liu D., Ni C., Wang Y., Zhu L. (2024). Review of serrated chip characteristics and formation mechanism from conventional to additively manufactured titanium alloys. J. Alloys Compd..

[58] Montgomery D.C. (2012). Design and Analysis of Experiments.

[59] Kasim M.S., Che H.C.H., Ghani J.A., Hadi M.A., Izamshah R., Anand T.J.S., Mohamed S.B. (2016). Cost evaluation on performance of a PVD coated cutting tool during end-milling of Inconel 718 under MQL conditions. Trans. IMF.

[60] Noordin M.Y., Venkatesh V.C., Sharif S., Elting S., Abdullah A. (2004). Application of response surface methodology in describing the performance of coated carbide tools when turning AISI 1045 steel. J. Mater. Process. Technol..

[61] Burnham K.P., Anderson D.R. (2004). Multimodel Inference: Understanding AIC and BIC in Model Selection. Sociol. Methods Res..

[62] Zhang G., Zhang J., Fan G., Xu C., Du J. (2023). The effect of chip formation on the cutting force and tool wear in high-speed milling Inconel 718. Int. J. Adv. Manuf. Technol..

[63] Li A., Zhao J., Hou G. (2017). Effect of cutting speed on chip formation and wear mechanisms of coated carbide tools when ultra-high-speed face milling titanium alloy Ti-6Al-4V. Adv. Mech. Eng..

